# Do Privacy Concerns About Social Robots Affect Use Intentions? Evidence From an Experimental Vignette Study

**DOI:** 10.3389/frobt.2021.627958

**Published:** 2021-04-26

**Authors:** Christoph Lutz, Aurelia Tamò-Larrieux

**Affiliations:** ^1^Nordic Centre for Internet and Society, BI Norwegian Business School, Oslo, Norway; ^2^Institute for Work and Employment Research, University of St. Gallen, St. Gallen, Switzerland

**Keywords:** social robots, privacy, trust, social influence, privacy paradox, survey

## Abstract

While the privacy implications of social robots have been increasingly discussed and privacy-sensitive robotics is becoming a research field within human–robot interaction, little empirical research has investigated privacy concerns about robots and the effect they have on behavioral intentions. To address this gap, we present the results of an experimental vignette study that includes antecedents from the privacy, robotics, technology adoption, and trust literature. Using linear regression analysis, with the privacy-invasiveness of a fictional but realistic robot as the key manipulation, we show that privacy concerns affect use intention significantly and negatively. Compared with earlier work done through a survey, where we found a robot privacy paradox, the experimental vignette approach allows for a more realistic and tangible assessment of respondents' concerns and behavioral intentions, showing how potential robot users take into account privacy as consideration for future behavior. We contextualize our findings within broader debates on privacy and data protection with smart technologies.

## Introduction

With the increasing interaction among humans and social robots (Fong et al., 2003; Gupta, 2015; Van den Berg, 2016), research on the benefits and concerns of close human–machine interaction has emerged. A field of research that has gained traction in recent years describes the privacy implications of social robots (cf. for an overview Lutz et al., 2019). This topic is particularly pressing because social robots tend to exhibit greater mobility, social presence, and autonomy than static devices (Calo, 2012; Kaminski, 2015; Lutz and Tamò, 2015, 2018; Sedenberg et al., 2016; Kaminski et al., 2017; Rueben et al., 2017a, 2018; Fosch-Villaronga et al., 2020). While research on privacy and social robotics has largely remained conceptual and has taken a critical approach to the data processing and privacy implications of social robots, a few studies provide quantitative evidence on the privacy concerns and implications of social robots (Lutz et al., 2019). However, initial survey-based studies have analyzed the existence of a robot privacy paradox (Lutz and Tamò-Larrieux, 2020), the trust implications of social robots (Alaiad and Zhou, 2014), as well as general attitudes toward them (Eurobarometer, 2012; Liang and Lee, 2017).

In this article, we aim to deepen our understanding of privacy in the context of social robots. We therefore present the results of an experimental vignette survey that assessed non-experts' privacy concerns about social robots and how these concerns affect use intention. The findings indicate that privacy matters. Individuals who are exposed to a more privacy-friendly robot, with the same functionality as a non-privacy-friendly robot, have significantly higher use intentions, even after controlling for relevant variables such as demographics, trusting beliefs, social influence, and general opinions about robots. Thus, our study furthers knowledge in the area of privacy-sensitive robotics (Rueben et al., 2018).

We start by describing the term “privacy” and point to a rich literature on the topic of privacy in the context of social robots. The literature review calls for a holistic understanding of the concept of privacy and embeds the topic in the human–robot interaction literature. We then describe the research model for the empirical study. An overview of the research method, including the sample, data analysis approach, and measurement, is followed by a description of the results. Subsequently, we discuss the findings, address the limitations of our approach, and contextualize the results.

## Literature Review

### Social Robots and Privacy Concerns

The introduction of new technologies has, throughout history, triggered a response in privacy scholarship (Warren and Brandeis, 1890; Calo, 2012; Finn et al., 2013). We can thus rely on a rich academic tradition of privacy scholarship when analyzing the privacy implications and concerns of social robots (Warren and Brandeis, 1890; Westin, 1967; Altman, 1975; Bygrave, 2002; Solove, 2008; Finn et al., 2013; Kaminski, 2015; Koops et al., 2016; Kaminski et al., 2017). While these discussions have had strong roots in the legal field, privacy research has become a multidisciplinary topic with various disciplines—from communication, computer science, psychology, sociology, and economy—collaborating with each other (Pavlou, 2011). This multitude of perspectives is very much welcome, yet also shows that defining a common notion of what privacy is remains difficult if not impossible (Solove, 2008). The difficulty arises not only out of the multitude of perspectives but also due to subjective and cultural differences and perceptions on privacy (Krasnova et al., 2012; Trepte et al., 2017). The cultural differences result also in different legal approaches of protecting informational privacy, with international agreements shaping their material and territorial scopes (Greenleaf, 2014; Greenleaf and Cottier, 2020).

Nonetheless, useful privacy categorizations and classifications exist. It is interesting to note that the literature conceptualizing privacy has often looked backward, describing how new technologies impact private and social life and finding remedies to address them (e.g., Warren and Brandeis, 1890). Newer scholarship (notably: Finn et al., 2013; Koops et al., 2016) provides more forward-looking frameworks by elaborating on the impact of newer technologies. These frameworks or taxonomies build upon the rich Western privacy literature. One important dimension here is the idea of “zones,” i.e., differentiating between more personal zones and more public ones (Koops et al., 2016). While the dichotomy between private and public spheres has been criticized in light of the increased pervasiveness of technology (Nissenbaum, 2004; Rouvroy, 2008; Acharya, 2015), different dimensions of privacy have been proposed (Rueben et al., 2017a; Lutz et al., 2019). One dimension deals with physical privacy concerns as the concerns relating to an individual's personal space (Finn et al., 2013). Such an understanding of privacy was already propagated by Warren and Brandeis (1890) and revolves around “physical access to an individual and/or the individual's surroundings and private space” (Smith et al., 2011, p. 990). Physical privacy concerns become especially apparent with the use of social robots at home due to the robot's ability to enter (uninvited) into private spaces (e.g., bathrooms, bedrooms) (Calo, 2012). However, new technologies, such as genetic codes and smart health tracking technologies (e.g., pills), have resulted in stronger demands for physical privacy. Proposals include the privacy of the person, which includes “the right to keep body functions and body characteristics private” (Finn et al., 2013, p. 8).

A second key dimension revolves around informational privacy concerns (Smith et al., 2011). At its core, informational privacy should enable individuals to have control about their information (Westin, 1967), thereby reducing institutional privacy threats by data-processing institutions (e.g., robot manufacturers, government agencies, and third parties such as data brokers or cloud providers) as well as social threats occurring by the processing of information by private individuals (e.g., familiar users, hackers) (Raynes-Goldie, 2010; Young and Quan-Haase, 2013). These aspects point to a core concern, namely, surveillance enabled by social robots that are equipped with innovative sensors and processors, enabling greater observation and profiling of individuals (Calo, 2012). In light of these technological changes, Koops et al. (2016) call for intellectual, decisional, associational, and behavioral privacy to ensure the self-development of individuals. Similarly, Finn et al. (2013) include in their seven types of privacy at least three types that are linked to informational privacy concerns, such as the privacy of personal behaviors and actions (including the revelation of sensitive habits and sexual orientation), the privacy of communication, and the privacy of data and images. All these types of information can be collected or disseminated through social robots. Similarly, and the reason why informational privacy concerns are closely tied to the ones mentioned below under boundary management, emerging technologies such as social robots will likely impact a user's privacy of thought and feelings (Finn et al., 2013). In addition, the way automated decision-making systems classify information about individuals and reach decisions (by correlations and pattern finding) affects a new class of privacy, namely, privacy of associations (Finn et al., 2013).

Closely tied are boundary management approaches, understanding privacy as a “selective control of access to the self or to one's group” (Altman, 1975, p. 18). This understanding of privacy as boundary management (Petronio, 2002) links back the discussion to the physical privacy concerns mentioned. However, boundary management approaches must be understood more broadly than pure “freedom from” and physical protection claims (Koops et al., 2016), as they put individuals and their agency to make own life choices at the center about when their privacy is unreasonably constrained (Carnevale, 2016). Agency requires understanding how information within social robots and various stakeholders is shared. In addition, research building on the boundary management literature indicates that the design of smart environments (e.g., setting of sensors and cameras), including ones with acting social robots in homes, impacts how these data-processing devices are perceived and privacy boundaries are negotiated (Schulz and Herstad, 2018; Schulz et al., 2018). The boundary management negotiations are highly dependent on the affordances of technologies (e.g., ability to turn sensors on and off) and the visibility of certain functionalities (e.g., surveillance through camera). Moreover, the anthropomorphic or zoomorphic effect of social robots (Fong et al., 2003; Weiss et al., 2009; Darling, 2016) may increase the pervasiveness of social robots (Turkle, 2011) and the bonding between individual and robot may inhibit rational and privacy-oriented considerations by individuals (Syrdal et al., 2007; Calo, 2012).

### Previous Research on Privacy and Social Robots

While there is a rich literature on human–robot interaction across disciplines (for an overview, see Baxter et al., 2016), research on privacy and social robots is still a comparatively nascent field. Early empirical studies on privacy concerns in the context of social robots have explored by means of qualitative interviews how individuals perceive the use of social robots in the work environment (e.g., Snackbot, see Lee et al., 2011). The study of Lee et al. (2011) revealed that most participants did not understand what data categories Snackbot collected and failed to differentiate between sensed data (“what the robot sees/hears”) and inferred information (“what the robot knows”, p. 182). Moreover, the anthropomorphic shape of Snackbot sometimes misled the participants' notion of the capabilities of the robot to record information (e.g., participants did not consider the ability of the robot to sense objects behind it).

Other empirical research has focused on concern related to information disclosure in human–robot interactions. For instance, in one study, participants stated that they overcame their fear of robots storing and accessing sensitive information about them because such processing activities were necessary (and thus tolerated) in order to benefit from the social robot's functions (Syrdal et al., 2007).

Other studies analyzed the privacy-utility tradeoff further, for instance, in the domain of teleoperated robots (Butler et al., 2015; Krupp et al., 2017). Butler et al. (2015) explored how, by means of visual filters, the privacy concerns of individuals can be reduced, and the benefits of teleoperated robots can still be reaped. Krupp et al. (2017) used focus groups to identify salient privacy concerns about telepresence robot. They found that informational concerns were most strongly discussed (106 occurrences of the theme in coding). However, physical concerns also received high attention with 60 occurrences. Social and psychological privacy, by contrast, received far less attention (both 16 occurrences). In addition, the study found important emerging categories that were sometimes understood in privacy terms, for example, marketing and theft. Other studies on home telepresence robots have studied how the framing of human–robot interaction and presentation of robot actions within a home by means of short video excerpts affects individual's privacy responses toward the robot (Rueben et al., 2017b). Rueben et al. (2017b) demonstrate the impact of what the authors call “contextual frames” on individuals' privacy judgments.

Furthermore, we see a growing, interdisciplinary interest in research about the privacy implications of social robots, with an uptick in publications across disciplines since 2015 (Lutz et al., 2019). To bridge the disciplinary gaps, expert workshop insights on currently under-addressed topics have led to the identification of interdisciplinary research needs (Rueben et al., 2018; Fosch-Villaronga et al., 2020; Kapeller et al., 2020) and have stipulated the emergence of new research fields, such as the field of privacy-sensitive robotics (Rueben et al., 2018). These workshops with experts across disciplines provide qualitative insights into the ethical, social, and legal implications of social (Rueben et al., 2018; Fosch-Villaronga et al., 2020) and wearable robots (Kapeller et al., 2020), pointing to the privacy-relevant issues to be tackled in the future. Privacy-related aspects include data privacy and transparency, deception and manipulation, agency and control, accountability, as well as trust, and recommendations on how to address them have been developed, for example, increased control and transparency requirements and the prohibition of data collection in certain instances.

Larger-scale quantitative studies, such as general population survey assessing citizens' attitudes and concerns toward robots, exist as well (e.g., Eurobarometer, 2012, 2015; Madden and Rainie, 2015). In the European Union (EU), the general attitudes toward robots are positive (64%) even though many fear that robots will take away jobs and alter the current labor market (70%). Interestingly, citizens in the EU did express some uneasiness with the idea of robotic companionship for elderly and surgical robots; yet the Eurobarometer (2015) did not link these feelings/responses to potential privacy concerns.

Overall, though, there seems to be a lack of empirical studies that assess the privacy concerns of social robots, especially with a quantitative approach (Lutz et al., 2019). Empirical research would prove effective to better understand the validity of theoretical knowledge on privacy. Moreover, empirical research can add a non-expert view on commonly theorized issues and, thus, take into account a more thorough perspective, potentially helping to shape responsible adoption in the future.

Our current study builds on earlier research that used a survey to test the privacy paradox among non-experts (Lutz and Tamò-Larrieux, 2020). This study found evidence for a robot privacy paradox, where users revealed privacy concerns (different levels, depending on the privacy type), but these concerns were not significantly correlated to robot use intentions, even after controlling for salient control variables such as expected benefits, social influence, scientific knowledge, and trust. Following up on this work, we aimed at a test that allowed to identify the role of privacy concerns less generally and more specifically. Thus, in contrast to the aforementioned study, our work here asks for privacy concerns about a fictional but concrete social robot, rather than social robots more broadly. The chosen method of an experimental vignette survey thus provides a more realistic test of the relationship between privacy concerns and robot use intentions.

### Privacy and Trust

The intricacies between privacy and trust is a complex phenomenon (Richards and Hartzog, 2016; Waldman, 2018). The abovementioned control and boundary-management functions of privacy enable interpersonal relationships that are built upon trust and trusting beliefs (Westin, 1967). At the same time, from an institutional perspective, companies including manufacturers of social robots might be incentivized to promote consumer trust by means of enhanced privacy features, linking privacy and trust via an economic element (Hartzog, 2018; Tamò-Larrieux, 2018). The importance of privacy for trust has also been acknowledged in more recent policy papers and ethical guidelines (European Commission, 2018, 2020; Delcker, 2019). While these papers and guidelines focus on artificial intelligence (AI) and ways to promote trustworthy AI, many operations of social robots already today employ such technology. These strategic objectives for AI will thus influence the development of social robots.

The relationship between trust and automation is complex, and literature on the subject has emerged (Lee and See, 2004; Cheshire, 2011; Hoffman et al., 2013; Schaefer et al., 2016; Botsman, 2018). While the relational perspective on trust among humans often defines trust as “a psychological state comprising the intention to accept vulnerability based upon positive expectations of the intentions or behaviors of another” (Rousseau et al., 1998, p. 395), Botsman (2018) understands trust as a “confident relationship with the unknown” (p. 20). Similarly, Möllering (2001) identifies trust as a three-step mental process of expectation, interpretation, and suspension. Interaction with a social robot requires trust because private information is disclosed to the machine. Such a disclosure requires a favorable expectation of an outcome that is uncertain. Whether or not an individual interprets an outcome to be favorable relies on a mix of different elements, including rational and emotional ones, and finally, the individual must take what Möllering (2001, p. 414) calls a “leap of trust,” meaning that what an individual interpreted becomes accepted and the unknownable momentarily certain.

The literature on interpersonal trust can help to understand trusting beliefs among humans and social robots (or more broadly: automation). However, “trust in automation involves other factors that relate specifically to technology's limitations and foibles” such as its “reliability, validity, utility, robustness, and false-alarm rate” (Hoffman et al., 2013, p. 85). The capabilities of technologies (such as social robots) as well as their affordances (e.g., ability to control certain features, communication with a device) impact trust in automation overall (Schaefer et al., 2016). What complicates the relationship further is that these technical features, which are continuously evolving with technological progress, are only one aspect in the broader calculus that impacts trust in the robot: Human factors (e.g., their personality, trust propensity, attitudes, etc.) and environmental ones (e.g., ways in which technologies are used within an environment, cultural notions) contribute to the full picture (Lee and See, 2004; Schaefer et al., 2016). Moreover, neither trust nor automation is static, but constantly evolving with new experiences. Capturing trust and trusting believes can thus only be done via proxy and illustrates a specific point in time and interaction with one technology.

With respect to trust and privacy in the context of social robots, an interesting field has emerged that analyzed situations in which users trust (Aroyo et al., 2018; Kundinger et al., 2019) and overtrust social robots (Booth et al., 2017; Borenstein et al., 2018; Wagner et al., 2018). Overtrust has an implication for privacy, as it indicates a tendency to allow physical, social, and informational constraints to be crossed. Overtrust is defined as “a situation in which a person misunderstands the risk associated with an action because the person either underestimates the loss associated with a trust violation; underestimates the chance the robot will make such a mistake; or both” (Wagner et al., 2018, p. 22). Thus, the topic of overtrust is closely linked to the one of deception, which—as mentioned above—multiple expert workshops have pointed to with a call for future, interdisciplinary research in the field. These aspects tie back the discussion on a political level, where the promotion and development of trustworthy technology is at the forefront of European policymakers' agenda (European Commission, 2018, 2020).

## Model and Theoretical Development

Behavioral intentions to use a social robot is the key dependent construct in this study. We used behavioral intentions, rather than actual or reported behavior, because of the topic of the study and nature of the data collection. We expected that few respondents had themselves interacted with a social robot. Thus, behavioral assessments would be unevenly distributed, less reliable, and less appropriate for the statistical analysis. Naturally, the reliance on behavioral intentions as the dependent construct, rather than actual behavior, prevents the test of the privacy paradox in the narrow sense. The privacy paradox is generally understood as the divergence between attitudes and behavior when it comes to privacy (Dienlin and Trepte, 2015; Kokolakis, 2017). It has evoked substantial research interest, especially in the context of digital and social media, as shown in the meta-analysis by Baruh et al. (2017), which included 166 studies with more than 75,000 participants across 34 countries in total. Their meta-analysis also discussed the distinction between behavioral intentions and actual behavior as the outcome variable in research on the privacy paradox. A considerable number of studies (about half of all the effects) in the meta-analysis relied on behavioral intentions, rather than actual behavior, as the outcome variable and the authors found similar effects for intentions and behavior: “[T]here were no significant differences between studies investigating behavioral intentions vs. behavior regarding use of online services […] and use of SNSs” (p. 39). The same was true for privacy protection intentions vs. privacy protection behavior as the outcome. Only for sharing information as the dependent variable, behavior and intentions behaved differently, with the effects for intentions being stronger than for behavior. Thus, based on the meta-analysis, two conclusions can be drawn. First, intentions is a frequently used dependent construct in privacy paradox, although it does not align with the commonly accepted definition. Second, intentions and behavior are similarly affected by privacy concerns. Taken together, and in conjunction with the empirical constraints of studying behavior in the context of social robots through a survey-based study, we deem it justifiable to look at intentions, rather than behavior.

In our research model, there are several attitudinal constructs to predict behavioral intentions: trust, privacy concerns, perceived benefits of robots, and scientific interest. We will discuss each of these factors in turn.

Trust, and specifically trusting beliefs, should be associated with robot use intentions. Trusting beliefs can be differentiated into specific dimensions such as integrity, benevolence, and ability (McKnight et al., 2002). Thus, the trustor must assess the trustee as honest, benevolent, and competent in order to form trust. If this is the case, individuals are more likely to develop trusting intentions, which will, in turn, lead to trusting behavior, for example, the use of a new technology. Trusting beliefs, rather than, for example, Möllering's (2001) leap of trust approach, are used because they are easier to measure. Based on the trust literature and trust theory, we propose the following hypothesis.

**H1**: *Trusting beliefs in a robot have a positive effect on social robot use intentions*.

Citizens need to overcome certain concerns to start using social robots voluntarily, privacy concerns being an important type. If the privacy risks of a social robot are thought to be high, we expect lower levels of adoption intention. However, ample research on self-disclosure and privacy in online contexts has shown that privacy attitudes—including concerns—often do not match privacy behavior (Kokolakis, 2017). Although they are concerned about their privacy, many users of digital services disclose sensitive information and do not protect their privacy adequately, for example, by choosing restrictive privacy settings. This divergence between attitudes and behavior is captured by the privacy paradox (Barnes, 2006). As discussed above, empirical studies that look at intentions, rather than behavior, are often also framed within a privacy paradox framework. In a way, we can interpret this understanding as a widened and broad take on the privacy paradox. To date, the empirical evidence on the privacy paradox—both in a strict and broad sense—is mixed. Many studies have identified a privacy paradox, but a considerable number of studies, especially newer ones, found significant effects between privacy attitudes and behavior or intentions, thus rejecting the privacy paradox. Kokolakis (2017) provides a systematic review of this literature, showing how the empirical evidence is inconclusive. Baruh et al. (2017) noted the absence of the paradox (i.e., there are small but significant effects between privacy attitudes and privacy-related behavior or intentions). However, their study also suggested that contexts matters because for social network sites, the privacy paradox seems to hold. In light of the emerging nature of social robots and low adoption rates, we expect that privacy concerns have a significant and negative effect on robot use intentions.

**H2**: *Privacy concerns about a robot have a negative effect on social robot use intentions*.

In the literature on the privacy paradox, different theoretical explanations for the paradox can be differentiated (Hoffmann et al., 2016). However, the privacy calculus has emerged as the dominant explanation (Dinev and Hart, 2006). Within this approach, users weigh the benefits and risks of a technology against each other and if the former outweigh the later, they will start or keep using the technology. A rich literature exists that analyzes the perceived risks and benefits of social network sites and elaborates on the privacy calculus in this context (Dienlin and Metzger, 2016; Trepte et al., 2017). This literature highlights the influence of cultural norms on privacy calculations (Krasnova et al., 2012; Trepte et al., 2017), showing how the privacy calculus is not a purely rational process but heavily influenced by cultural and psychological default positions. Moreover, the framing of privacy concerns and sharing benefits will likely affect use intentions. Coopamootoo and Groß (2017) found that privacy attitudes and sharing attitudes differed significantly in terms of emotional connotation. Privacy attitudes related to fear, bringing up actors with a negative connotation such as hackers and data collectors (e.g., Google). By contrast, sharing attitudes related to joy, bringing up actors with a positive connotation such as family and friends. Thus, whether individuals are in a privacy mindset or a sharing mindset might result in different behaviors. Applied to social robots, depending on the framing of the discussion and if this technology is seen as very useful and benefitting their personal lives (i.e., sharing attitudes are prioritized over privacy attitudes), individuals will have higher use intentions. On the other hand, if a social robot is framed more in privacy terms, individuals will have lower use intentions. Given theories such as the theory of planned behavior (TPB; Ajzen, 1991) and previous research (Alaiad and Zhou, 2014), we expect that perceived benefits exert a positive influence on robot use intentions.

**H3**: *Perceived benefits of social robots have a positive effect on social robot use intentions*.

In TPB, social influence is an important antecedent of behavioral intention (McEachan et al., 2011). Likewise, technology adoption approaches, for example, the technology acceptance model and the unified theory of acceptance and use of technology (UTAUT) highlight the key role of social factors people's adoption decisions (Venkatesh and Morris, 2000; Venkatesh et al., 2003). In these theories, social influence increases behavioral intentions to adopt a new technology. As a not yet widely adopted technology, social robots should drive use intentions when someone's social environment encourages or expects their use. Citizens that have more social robot-friendly networks should therefore have higher intentions to use them.

**H4**: *Social influence has a positive effect on social robot use intentions*.

Scientific interest was included as a control variable. Citizens who are more scientifically interested tend to be more up-to-date with recent technological developments, including those that pertain to social robots. Since social robots are still not widely adopted, we consider scientific interest as a proxy for knowledge and awareness of social robots—and technology skills with social robots. Extant research has shown that (digital) technology skills vary by education (Van Deursen and Van Dijk, 2011; De Boer et al., 2020). Based on De Boer et al. (2020) study about internet-of-things technologies, which share similarities with social robots, we expect technology skills with social robots to vary by education level as well. Given that scientific interest and technology skills are both shaped by someone's education, we think it is justifiable to use scientific interest as a proxy for technology skills with social robots, particularly in a situation where individuals do not have experience with the technology itself (i.e., they do not own a robot). Scientifically interested citizens should be able to assess the benefits and risks of the technology more closely, including the privacy risks. They might also be more technologically open minded and curious. Using diffusion of innovation theory as a conceptual basis (Rogers, 2003), citizens interested in scientific development should have higher behavioral intentions to use novel technologies, including social robots. By including scientific interest, we also follow existing survey-based studies (Eurobarometer, 2012).

**H5**: *Scientific interest has a positive effect on social robot use intentions*.

## Methods

### Sample

The experimental vignette study was conducted in December 2018, in the form of a randomized survey with two conditions: high privacy risks and low privacy risks. We programmed the survey in Qualtrics and relied on MTurk for the participant recruitment, surveying respondents located in the United States (see Aguinis et al., 2020 for more information on MTurk as a data source). The average completion time was 8 min and participants were compensated with 1.25 US dollars, leading to an average hourly wage of 9.5 US dollars. We aimed for a sample of 300 participants–150 per condition—and in the end, 298 respondents completed the study. Because they failed at least one of two attention checks, six individuals were eliminated from further analysis, leaving us with a final sample of 292. The average age in the final sample is 35 years old (median = 33 years; SD = 9.5 years). One hundred fourteen respondents identify as female (39%), 177 as male (60.5%), and one person prefers not to say (0.5%). The sample is relatively educated, with 16% having high school as their highest degree, 36% some college, 41% a Bachelor, 6.5% a Master, and 0.5% a Doctorate or Other.

### Measurement and Data Analysis

To test our hypotheses, we used a people paper study (Aguinis and Bradley, 2014) with a between-subjects design and with a manipulation of privacy risks into a high and low condition. Participants were randomly assigned into either the high or low privacy risk scenario. The vignette described a fictional social robot called MIMO. MIMO is portrayed as an affordable companion robot that offers useful functionality. In both conditions, the respondents saw the same introductory paragraph describing MIMO's general functionality and purpose. However, the next two paragraphs differed between the two conditions. In the first and low privacy risk scenario, MIMO is e-privacy certified and fully complies with current US and European privacy laws. MIMO tends to have privacy-by-default settings in this scenario and fewer privacy-invasive capabilities than in the high privacy risk scenario. Moreover, the data MIMO collects is stored more securely and locally. By contrast, MIMO in the high privacy risk scenario is not e-privacy certified and does not comply with US and European privacy law (compliance with privacy laws is not a condition for market entrance but suppliers that violate privacy laws risk facing steep fines, Newlands et al., 2020). In this scenario, MIMO is more privacy-invasive, for example, by being switched on by default and performing additional analyses on the users' voice. Moreover, MIMO has worse security in this scenario. The two privacy risk scenarios are shown in Supplementary Figures 1 and 2 in Supplementary Material. We focused strongly on informational privacy for these scenarios but included elements of other privacy types as well. For the formulation of the scenarios, we followed established privacy conceptualizations and measurements (e.g., Malhotra et al., 2004; Stutzman et al., 2011), intending that the low-risk scenario would result in lower privacy concern scores on these scales and that the high privacy risk scenario would result in higher scores. As a manipulation check, participants responded to 16 privacy concern questions/items that can be grouped into four privacy concern types [Lutz and Tamò-Larrieux (2020) for more information on these four dimensions]. The manipulation checks indicated that the conditions clearly differentiated privacy concerns (Table 1).

**Table 1:** Manipulation check.

	**Low privacy risk condition**	**High privacy risk condition**	***t*-value**	**Sig**.	**Mean difference [confidence interval]**
Physical privacy concerns	2.02	2.61	5.01	0.00	0.59 [0.36, 0.83]
Institutional informational privacy concerns	3.14	4.22	8.15	0.00	1.08 [0.82, 1.34]
Social informational privacy concerns	2.66	3.89	9.18	0.00	1.23 [0.97, 1.49]
Overall privacy concerns	2.31	3.67	9.82	0.00	1.36 [1.09, 1.63]

*Arithmetic means are displayed for columns 2 and 3; 1–5 scales; N = 143 for low(er) privacy risk scenario and 149 for high(er) privacy risk scenario; Levene's test for equality of variances yields p-values > 0.05 for social, physical, and global privacy concerns, indicating equal variances assumed, but <0.05 for institutional privacy concerns; measurement of privacy concerns dimensions based on Lutz and Tamò (2015)*.

The privacy risk manipulations were entered into a regression as dummy variables (0—low privacy risk, 1—high privacy risk), and we used robot use intentions as the dependent variable. Principal component analysis was used to bundle all constructs with more than one item (i.e., robot use intentions, overall privacy concerns, trusting beliefs, social influence). All four constructs loaded neatly on one component and had high internal consistency. Cronbach's α was 0.96 for robot use intentions, 0.94 for overall privacy concerns, 0.92 for trusting beliefs, and 0.91 for social influence. No significant demographic differences in age (*t* = 0.76, *p* = 0.45), education (*t* = 0.11, *p* = 0.92), and gender (Chi-Square = 2.15, *p* = 0.34) exist between the respondents in the low and high privacy risk scenarios.

We used the measures from Lutz and Tamò-Larrieux (2020) to assess social robot use intention, social influence, trust, and scientific interest but slightly adjusted them to make the connection to the vignettes and MIMO. More specifically, the prompts at times reminded the respondents to think of the social robot described in the scenario and the items referred to this specific social robot rather than robots in general (e.g., for trusting beliefs, two sample items were “*I believe that such a robot acts in my best interest.”* and “*Overall, such a robot is a capable and proficient service provider.”*). For perceived benefits, we used a more succinct measurement with only one item based on the Eurobarometer (2012) survey. The item assessed respondents' opinion about social robots in general terms and had four response options: very negative, fairly negative, fairly positive, and very positive. The full questionnaire used is shown in Supplementary Material.

To test the hypotheses, we conducted a linear regression analysis in Stata (v.15), using the “robust” option for heteroscedasticity-corrected standard errors. We also tested for colinearity, and the largest variance inflation factors were 2.17 and 2.14 for the education categories “some college” and “Bachelor's degree,” respectively, indicating the absence of severe colinearity. Privacy concerns were entered as an independent dummy variable based on the condition (high privacy risk scenario vs. low privacy risk scenario).

## Results

Table 2 displays the results of the linear regression analysis. Trusting beliefs in the robot have a significant and positive effect on robot use intentions. The null hypothesis is therefore rejected, offering some support for H1. Privacy concerns have a significant and negative effect on social robot use intention, rejecting the null hypothesis and offering some support for H2. Controlling for demographic characteristics, trust, benefits/general opinion toward social robots, social influence, and scientific interest, respondents in the high-risk scenario score two thirds of a point lower (on a five-point scale) in their intention to adopt the social robot. Perceived benefits, in the form of general opinions about robots, have a significant and positive effect on robot use intentions, refuting the null hypothesis and offering some support for H3. Social influence affects robot use intentions significantly and positively. Thus, the null hypothesis is rejected for H4, and some support is found for this hypothesis. The more supportive someone's social environment toward robots, the higher the intentions to use such a robot. Finally, H5 is rejected as scientific interest does not influence robot use intentions significantly. The demographic predictors exert a limited influence on robot use intentions, but more educated users have somewhat higher use intentions.

**Table 2:** Regression of robot use intentions on demographics, privacy, trusting beliefs, general opinion/beliefs, social influence, and scientific interest.

	**Unstandardized coefficient (robust standard errors)**	**Beta**
Age	0.01 (0.01)	0.04
Gender (reference: female)		
Male	−0.03 (0.10)	−0.01
Other	−0.77^***^ (0.13)	−0.04
Education (reference: high School)		
Some college	0.2 (0.14)	0.07
Bachelor	0.19 (0.14)	0.07
Master	0.43^*^ (0.21)	0.08^*^
Doctor	0.78^***^ (0.25)	0.04^***^
Other	−0.02 (0.16)	0.00
Privacy risk condition (reference: low risk)	−0.65^***^ (0.11)	−0.25^***^
Trusting beliefs	0.29^***^ (0.07)	0.22^***^
General opinion/benefits	0.22^**^ (0.08)	0.12^**^
Social influence	0.54^***^ (0.06)	0.47^***^
Scientific interest	0.07 (0.10)	0.03
Constant	−0.47 (0.42)	

*N = 292; R^2^ = 0.62*;

*** *p < 0.001*,

** *p < 0.01*,

* *p < 0.05, no star, not statistically significant. A Bonferroni correction that assumes a p-value threshold of 0.05 would result in a corrected statistical significance threshold of 0.00625 (0.05/8), since there are eight predictor variables, five from the hypotheses, and three control variables. Education: Master is the only effect that becomes insignificant after such a correction. All other significant predictors have p-values below 0.00625*.

Overall, four out of the five hypotheses tend to be supported, and one is rejected. Importantly, the main hypothesis about the privacy paradox (H2) was not rejected. We are able to explain 62 percent of the variance in intention to use the fictional social robot with our independent variables.

## Discussion and Conclusion

Previous research on privacy in digital contexts has detected a privacy paradox between users' privacy attitudes and their behaviors as well as between privacy attitudes and intentions. Users report high levels of privacy concerns but exhibit behavior that could be interpreted divergently, such as high levels of disclosure of personal information and low levels of privacy protection (Chen and Rea, 2004; Milne et al., 2009). While the individualistic notion of the privacy paradox and privacy self-management is increasingly contested (e.g., Obar, 2015; Lutz et al., 2020), we, nevertheless, took the privacy paradox as a useful starting for investigating social robots as an emerging but not yet widely adopted technology. Following up on earlier work, where we had tested the privacy paradox for social robots more generally and indeed found evidence for a privacy paradox (Lutz and Tamò-Larrieux, 2020), we wanted to check whether the privacy paradox between privacy concerns and intentions holds when confronted with a concrete robot. We, thus, opted for an experimental vignette study as a middle ground between a lab study, which is costly and time intensive, and a more generic survey. The experimental vignette study allows testing the relationship between privacy concerns and robot use intentions in a causal sense and is more tangible than a general survey.

We found that the privacy manipulation had a relatively pronounced effect on robot use intentions. Respondents in the more invasive scenario were significantly less likely to be willing to use such a robot, controlling for a range of predictors. Trusting beliefs, social influence, and general opinion about robots also influenced robot use intentions significantly—and positively.

Several theoretical and practical *implications* come with our findings. Importantly, we did not find a privacy paradox and instead identified a strong role of privacy invasiveness in affecting use intentions. This is in line with overview articles that looked at the privacy paradox more generally. For example, Baruh et al. (2017), in their meta-analysis of research on the privacy paradox, identified that, on aggregate, the privacy paradox does not hold, and there is, in fact, an association between privacy concerns and privacy-related behavior as well as intentions. Similarly, Kokolakis (2017), in a systematic literature view on the privacy paradox, discussed a temporal trajectory with newer studies increasingly refuting the privacy paradox. Our research indicates that when individuals are confronted with concrete privacy-invasive technologies, they do take privacy into consideration. Thus, privacy matters—and will matter—for social robots (Rueben et al., 2018). However, a limitation of our study is that we used intentions rather than actual behavior as the dependent variable, due to the practical constraints of recruiting social robot owners with a general survey and constraints in doing a lab study. As discussed, the focus on intentions aligns with other research on the privacy paradox (see Baruh et al., 2017) but does not follow the original conceptualization of the privacy paradox as a divergence between attitudes and behavior. Thus, future research could confront individuals with actual robots that vary in privacy friendliness and test whether individuals use them differently in a controlled setting (of course making sure that no ethical boundaries are crossed and that users' privacy is not actually violated within the study). Research could also investigate the privacy paradox for adjacent technologies such as smart speakers and smart toys, which share similarities with social robots but are more widely adopted and therefore easier to sample for (Peter et al., 2019; Lutz and Newlands, 2021). Moreover, the privacy aspects were quite prominent in our vignettes. When deciding about purchasing a social robot in real life, potential users will probably not have the same concise privacy information available as in the study. With this in mind—and taking the literature on the privacy calculus and cultural differences into account (Krasnova et al., 2012; Trepte et al., 2017) as well as the one highlighting the limitations of rational decisions with respect to privacy (e.g., Acquisti and Grossklags, 2005)—it remains to be seen how privacy-friendly design of social robots impacts the willingness of users to buy and engage with them. Overall, as indicated by the literature on cultural privacy preferences, we assume that our results with respect to the use intention provided with substantial information on the privacy risks of devices would change depending on the dominant culture of a test group.

Another important finding is that trusting beliefs affect social robot use intention positively. Thus, individuals take the trustworthiness of a social robot into consideration when considering using it. We have discussed in the literature review how this can have ambivalent consequences, especially if users trust social robots too much (Booth et al., 2017; Borenstein et al., 2018; Wagner et al., 2018). Future research should explore the dynamics of trust and overtrust in social robots, and their connection to privacy. Such research is needed as overtrusting social robots might have serious privacy implications as overtrust leads to a tendency to allow physical, social, and informational boundaries to be crossed. Interdisciplinary research in this field should furthermore examine how deception by social robots influences privacy perceptions, use intentions, and trusting beliefs. Findings in those areas would further promote the policy objectives of the European Union, which aims at developing trustworthy technology (European Commission, 2018, 2020).

The support for H3 about a positive influence of perceived benefits/general opinion about robots points to the partly utilitarian nature of the technology. More positive opinions about robots will translate into higher use intentions. Future research could disentangle these opinions somewhat and investigate how positive or negative opinions are formed based on different factors such as media consumption, education, and technology attitudes more broadly. A limitation of our study was the single-item measurement of this construct. Future research should use more robust, multi-item scales to assess perceived benefits and general opinions about robots. Uses and gratifications would be a helpful theory to systematically develop perceived benefits (De Jong et al., 2019).

The finding that social influence has a positive effect on use intentions suggests that the use of social robots, as an emerging technology, depends heavily on someone's social environment. Thus, social robots have to be understood in context and their situatedness within certain social milieus (e.g., educated and tech-affine people) begs for further study, especially through observational and qualitative approaches. Our findings show that social norms are of crucial importance in the context of social robots. Robotics firms might want to invest in community management and word-of-mouth promotion to leverage this social influence.

Overall, our study suggests that privacy matters. Robotics firms should therefore take privacy sensitivity into consideration as an important design factor. If privacy is neglected and privacy invasions occur, the media are quick to highlight these issues, as it happened when privacy norm violations with related technologies, such as smart speakers, occurred (Estes, 2018; Day et al., 2019a,b). Thus, robotics firms should construe privacy as a key part of their development philosophy and not as an afterthought. In Europe, this is legally mandated by the privacy-by-design and privacy-by-default principle established within the General Data Protection Regulation (GDPR). How the principle of privacy-by-design and privacy-by-default will impact the concrete design of social robots is still to be seen.

Robotics firms should be aware of the fact that consumers value privacy and consider it in their purchasing decisions when faced with tangible risks. In that sense, manufacturers might want to increase investments into privacy-sensitive robotics (Rueben et al., 2018). Not only should manufacturers develop social robots that are privacy friendly, but they should also communicate their privacy-protection efforts to potential customers in concise and transparent ways (Felzmann et al., 2019). Here too, the GDPR paves the way in Europe with a list of specific information duties that data controllers (i.e., entities determining what data are being processed for what purpose) must provide to the data subjects (i.e., the person affected by a data processing of a social robot and to whom the personal data being processed belongs to).

Aside from government strategy positions (e.g., European Commission, 2018, 2020), newer industry standards on “trustworthiness in artificial intelligence” (ISO/IEC TR 24028:2020) elaborate on approaches toward security and privacy in AI. Such self-regulatory standards show that also the industry has realized the need for a holistic and standardized manner to ensure trust in AI as well as AI-based products (e.g., social robots). It will be interesting to follow how the strategy positions of governments will shape the approaches of the industry through standardization efforts as well as upcoming legislation.

## Data Availability Statement

The raw data supporting the conclusions of this article will be made available by the authors upon request, without undue reservation.

## Ethics Statement

Ethical review and approval was not required for the study on human participants in accordance with the local legislation and institutional requirements. The patients/participants provided their written informed consent to participate in this study.

## Author Contributions

CL and AT-L were jointly responsible for coming up with the paper idea and the research design, carrying out the data collection, and writing the Introduction and passages within Discussion and Conclusion. CL was mostly responsible for the data analysis and writing of the sections Model and Theoretical Development, Methods, and Results. AT-L was mostly responsible for writing the Literature Review section and parts of the Discussion and Conclusion section. Both authors contributed to the article and approved the submitted version.

## Conflict of Interest

The authors declare that the research was conducted in the absence of any commercial or financial relationships that could be construed as a potential conflict of interest.

## Supplementary Material

Supplementary Figure 1 Experimental vignette for low privacy risk scenario (privacy-friendly robot).

Supplementary Figure 2 Experimental vignette for high privacy risk scenario (privacy-unfriendly robot).
