# Supplementary figures and images for: Do Privacy Concerns About Social Robots Affect Use Intentions? Evidence From an Experimental Vignette Study
Source: Front Robot AI. 2021 Apr 26;8:627958. doi: 10.3389/frobt.2021.627958 (PMC8110194; doi:10.3389/frobt.2021.627958)

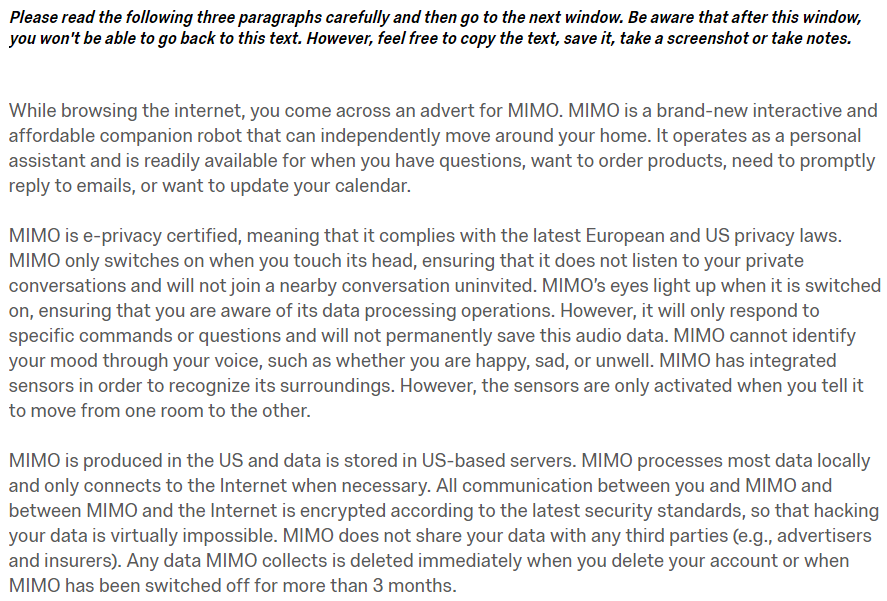

Supplement: Supplementary Figure 1 — Experimental vignette for low privacy risk scenario (privacy-friendly robot). [file Image_1.png]

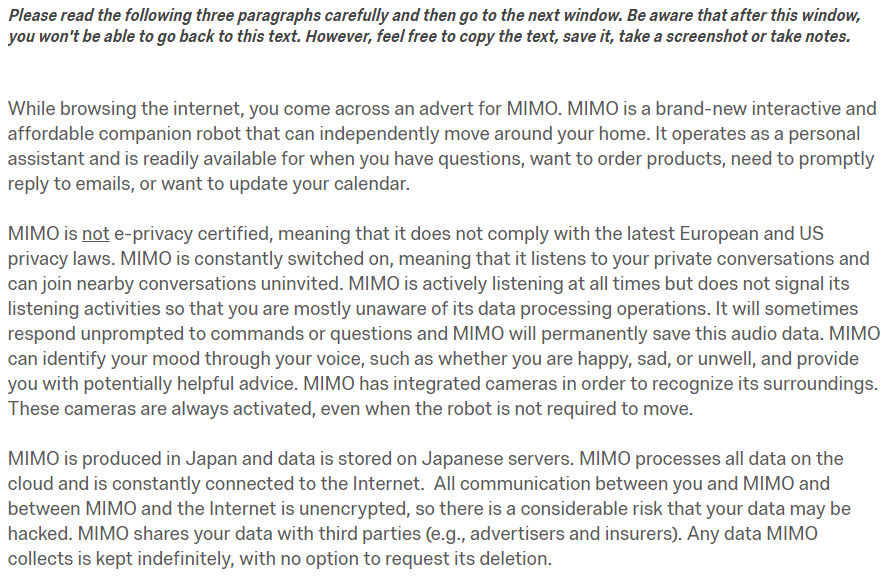

Supplement: Supplementary Figure 2 — Experimental vignette for high privacy risk scenario (privacy-unfriendly robot). [file Image_2.png]
