# Supplementary material for: Do Privacy Concerns About Social Robots Affect Use Intentions? Evidence From an Experimental Vignette Study
Source: Front Robot AI. 2021 Apr 26;8:627958. doi: 10.3389/frobt.2021.627958 (PMC8110194; doi:10.3389/frobt.2021.627958)
Supplement: Supplementary file 3 [file Table_1.docx]

**Questionnaire**

| Robot Use Intention  (based on Venkatesh et al., 2003) | *Please tell us how much you agree or disagree with the following statements.* |
| --- | --- |
|  | *I would very much like to have such a robot at home.* |
|  | *I would very much like to have such a robot at work.* |
|  | *I will probably use such a robot in the near future at home.* |
|  | *I will probably use such a robot in the near future at work.* |
|  | *I would recommend such a robot to other people.* |
| Trusting Beliefs  (based on McKnight et al., 2002) | ***Please tell us how much you agree or disagree with the following statements.*** |
|  | *I believe that such a robot acts in my best interest.* |
|  | *If I required help, such a robot would do their best to help me.* |
|  | *Such a robot performs its role of offering personal services really well.* |
|  | *Overall, such a robot is a capable and proficient service provider.* |
|  | *Such a robot would keep its commitments.* |
| Social Influence  (based on Venkatesh et al., 2003) | ***For the following statements, imagine you had a robot at home such as one of those shown in the pictures at the beginning of the survey. Please tell us how much you agree or disagree with the following statements.*** |
|  | *People who influence my behavior think I should use such a robot.* |
|  | *People who are important to me think that I should use such a robot.* |
|  | *In general, my friends have supported or would support the use of such a robot.* |
| Overall Privacy Concerns (used in regression)  (based on Malhotra et al., 2004) | ***Please tell us how much you agree or disagree with the following statements.*** |
|  | *Overall, I see a real threat to my privacy due to the robot.* |
|  | *I fear that something unpleasant can happen to me due to the presence of the robot.* |
|  | *I do not feel safe due to the presence of the robot.* |
|  | *Overall, I find it risky to have such a robot.* |
| Physical Privacy Concerns (used in manipulation check)  (based on Lutz & Tamò-Larrieux, 2020) | ***How concerned are you about the following potential privacy risks that could emerge when using such a robot?*** |
|  | *The robot damaging or dirtying my personal belongings (e.g., furniture)* |
|  | *The robot asking me personal questions* |
|  | *The robot snooping through my personal belongings (e.g., pictures)* |
|  | *The robot entering areas it shouldn't access (e.g., bedroom)* |
|  | *The robot using items it shouldn't use (e.g., bedclothes, pillows, personal hygiene products)* |
| Institutional Informational Privacy Concerns (used in manipulation check)  (based on Lutz & Tamò-Larrieux, 2020) | ***How concerned are you about the following potential privacy risks that could emerge when using such a robot?*** |
|  | *The manufacturer of the robot insufficiently protecting personal data* |
|  | *The manufacturer of the robot tracking and analyzing personal data* |
|  | *The manufacturer of the robot selling personal data to third parties* |
|  | *The manufacturer of the robot sharing personal data with government agencies* |
| Social Informational Privacy Concerns (used in manipulation check)  (based on Lutz & Tamò-Larrieux, 2020) | ***How concerned are you about the following potential privacy risks that could emerge when using such a robot?*** |
|  | *Other users engaging in identity theft through the robot* |
|  | *Other users hacking into the robot* |
|  | *Other users stalking me via the robot* |
| Perceived Benefits / General Opinion of Robots  (Eurobarometer 2012) | ***Generally speaking, what is your opinion of robots?*** Very positive; somewhat positive; fairly negative; somewhat negative |
| Scientific Interest  (Eurobarometer 2012) | ***Please tell us whether you are very interested, moderately interested or not at all interested in scientific discoveries and technological developments.*** |

Table note: We relied on 5-point Likert scales ranging from “strongly disagree” to “strongly agree” for all items, except for the single-item measures (see response options in cells) and privacy concerns. Here, respondents could assess their concern on a 5-point scale ranging from “no concern at all” (1) to “very high concern” (5).”

*Table A. Questionnaire and items used*
